# Yeast polysaccharide supplementation: impact on lactation, growth, immunity, and gut microbiota in Dezhou donkeys

**DOI:** 10.3389/fmicb.2023.1289371

**Published:** 2023-11-09

**Authors:** Bingjian Huang, Muhammad Zahoor Khan, Yinghui Chen, Huili Liang, Xiyan Kou, Xinrui Wang, Wei Ren, Changfa Wang, Zhenwei Zhang

**Affiliations:** ^1^Liaocheng Research Institute of Donkey High-Efficiency Breeding and Ecological Feeding, Liaocheng University, Liaocheng, China; ^2^College of Life Sciences, Liaocheng University, Liaocheng, China; ^3^Faculty of Veterinary and Animal Sciences, University of Agriculture Dera Ismail Khan, Dera Ismail Khan, Pakistan

**Keywords:** yeast polysaccharide, Dezhou donkeys, foals, lactational performance, growth, plasma metabolites, immune indices, gut microbiota

## Abstract

**Introduction:**

The Dezhou donkey, a prominent Chinese breed, is known for its remarkable size, rapid growth, and resilience to tough feeding conditions, and disease resistance. These traits are crucial in meeting the growing demand for Ejiao and donkey meat. Yeast polysaccharide (YPS), a functional polysaccharide complex known for its immune-enhancing and growth-promoting properties in livestock and poultry, remains relatively understudied in donkeys.

**Objectives:**

This study aimed to investigate the impact of YPS supplementation on lactating and growing Dezhou donkey jennies and foals.

**Materials and methods:**

Twelve 45-day-old Dezhou donkey foals and their jennies, matched for body weight and age, were randomly allocated to two dietary groups: a control group receiving a basal diet and an experimental group receiving the basal diet supplemented with 10 g/pen of YPS. The experiment was conducted over a 23-day period, during which donkey foals and lactating jennies were co-housed.

**Results and discussion:**

The findings revealed that YPS supplementation had no adverse effects on milk production or composition in Dezhou donkey jennies but significantly increased feed intake. Additionally, YPS was associated with increased plasma glucose and creatinine concentrations in foals, while tending to decrease alkaline phosphatase, white blood cell count, red blood cell count, and hemoglobin levels (*p* < 0.10). Immune indices demonstrated that YPS supplementation elevated the levels of immunoglobulin A (IgA) and immunoglobulin G (IgG) in jennies (*p* < 0.05) and increased complement component C4 concentrations in foals (*p* < 0.05). Moreover, YPS positively influenced the fecal microbiome, promoting the abundance of beneficial microorganisms such as *Lactobacillus* and *Prevotella* in donkey foals and *Terriporobacter* and *Cellulosilyticum* in jennies, all of which contribute to enhanced feed digestion. Additionally, YPS induced alterations in the plasma metabolome for both jennies and foals, with a predominant presence of lipids and lipid-like molecules. Notably, YPS increased the concentrations of specific lipid metabolites, including 13,14-Dihydro PGF2a, 2-Isopropylmalic acid, 2,3-Dinor-TXB2, Triterpenoids, Taurocholic acid, and 3b-Allotetrahydrocortisol, all of which are associated with improved animal growth.

**Conclusion:**

In conclusion, this study suggests that dietary supplementation of YPS enhances feed intake, boosts immunity by increasing immunoglobulin levels, stimulates the growth-promoting gut microbiota (*Lactobacillus* and *Prevotella*), and exerts no adverse effects on the metabolism of both Dezhou donkey jennies and foals.

## 1. Introduction

As a species with a storied history, the donkey’s role in human civilization has evolved over time, transitioning from a utilitarian work animal to an economically valuable asset capable of producing skin, meat, and milk (Wang et al., 2020). Among the diverse donkey breeds in China, the Dezhou donkey stands out as one of the five most significant (Seyiti and Kelimu, 2021). Presently, the primary breeding objective for the Dezhou donkey is to develop high-quality specimens renowned for their exceptional hide and meat yields. Concurrently, within the realm of animal nutrition, there is a growing emphasis on enhancing the hide and meat production of Dezhou donkeys (Liu et al., 2022; Wang et al., 2022).

The research on the impact of dietary polysaccharide additives on various aspects of animal physiology, including growth performance, immunity and anti-inflammatory and antioxidant status, has been extensively documented in recent years by multiple studies (Tan et al., 2017; Li et al., 2019; Li M. et al., 2022; Yu et al., 2022; Huang et al., 2023). Furthermore, a recent experimental trials conducted by Huang et al. specifically investigates the effects of dietary *Lycium barbarum* polysaccharides (LBP) on spotted sea bass (Huang et al., 2023). Their findings demonstrate that the inclusion of LBP in the diet can lead to improvements in growth, digestion, antioxidant capacity, and liver health. Notably, LBP supplementation effectively mitigates lipid metabolism disorders induced by high dietary lipid intake, which underscores its potential as a beneficial dietary component. Similarly, a study by Yu et al. explores the impact of dietary supplementation with *Taraxacum mongolicum* polysaccharide (TMP) on Jian carp (Yu et al., 2022). Their research highlights significant improvements in growth, digestive enzyme activity, immune response, and antioxidant status. Furthermore, the study elucidates the regulatory effects of TMP on the expression of key genes related to the NF-κB, Nrf2, and TOR signaling pathways that were associated with inflammatory and antioxidant regulation processes. Another noteworthy investigation by Li M. et al. focuses on the potential benefits of *Hippophae rhamnoides* polysaccharide (HRP) in mitigating inflammatory damage in intestinal porcine epithelial cells (IPEC-J2; Li M. et al., 2022). Their findings indicate that HRP exerts its protective effects by inhibiting the mitogen-activated protein kinase (MAPK)/nuclear factor kappa-B (NF-κB) signaling pathway activation, thus underscoring its anti-inflammatory properties. In addition to the above studies, Li M. et al. conducted research on the dietary *Allium mongolicum Regel* polysaccharide (AMRP) and its effects on *Channa argus* (Li et al., 2019). Their study demonstrates that AMRP supplementation significantly improves weight gain (WG) and specific growth rate (SGR) after 56 days of feeding trials. Importantly, AMRP is shown to prevent oxidative stress and inflammatory changes induced by lipopolysaccharide (LPS), which is achieved through enhancements in the antioxidant activity of superoxide dismutase (SOD), glutathione-S-transferase (GST), interleukin-1β (IL-1β), and tumor necrosis factor-α (TNF-α). In addition to the above mentioned key important functions of dietary polysaccharides additives, their positive impact on fecal microbiota and milk production performances has also been reported (Aung et al., 2019; Peng et al., 2020; Liang et al., 2021; Tian et al., 2022; Vlassopoulou et al., 2022; Zhang et al., 2022).

YPS emerges as a pivotal player in this endeavor, characterized as a macromolecular complex polysaccharide extracted from yeast cell walls, comprising distinct layers encompassing mannan, glycoprotein, and β-glucan layers (Kogan et al., 2008). Owing to its regulatory influence on various biological processes and metabolic pathways, YPS has garnered increasing attention within both human and animal research domains. Specifically, in the realm of animal husbandry, YPS has demonstrated its capacity to bolster growth performance, bolster immune function, foster optimal gut health, and curtail mortality rates (Eicher et al., 2010; Conway et al., 2022). Previous studies have yielded comparable results, as evidenced by increased average daily gains and enhanced feed conversion rates in holstein bulls following YPS supplementation (Ma et al., 2015). Furthermore, research indicates that the inclusion of yeast polysaccharides in diets significantly enhances milk production in dairy cows, albeit without substantial impact on milk fat, lactose, or milk protein (Nocek et al., 2011). Notably, YPS also contributes to improved milk quality by reducing milk somatic cell counts and elevating immunoglobulin levels in colostrum (Xia et al., 2021). Furthermore, YPS has been shown to augment the populations of beneficial intestinal flora, thereby ameliorating gut health in various animal species, including pigs and chickens (de los Solis Santos et al., 2007; Stuyven et al., 2009).

Hence, yeast polysaccharide (YPS) emerges as a multifaceted functional polysaccharide complex with a track record of bolstering immunity, enhancing intestinal microbiota, and elevating growth performance in livestock and poultry. However, to the best of our knowledge, the effects of YPS impacts on immunity, fecal microbiota, lactational and growth performance of donkeys has got limited attention so far. Thus, the current study endeavors to bridge this knowledge gap by investigating the impact of YPS supplementation on lactating and growth performance, plasma profiles, immune indices, and fecal microbiome composition in Dezhou donkey jennies and foals. The forthcoming results can provide valuable insights that could inform the practical application of YPS in enhancing productivity and immunity within the donkey population.

## 2. Materials and methods

### 2.1. Ethical statement

The utilization of animals in this study adhered to rigorous ethical standards and was formally approved by the Animal Ethics Committee of Liaocheng University under the reference number LC2019-1. All procedures conducted in this study were in strict compliance with established animal welfare protocols.

### 2.2. Animals and diets

Twelve robust 1.5-month-old donkey foals, exhibiting comparable initial size characteristics, were meticulously selected alongside their lactating jennies. These subjects were methodically divided into two distinct groups, each comprising 6 replicates, with a single mother-offspring pair per replicate. Group I was administered the basal diet (as outlined in Table 1), whereas Group II received the basal diet supplemented with 10 g/ (jenny and their foal • d) of yeast polysaccharides. The trial period spanned 23 days, and the source of the yeast polysaccharides was Angel Yeast Co., Ltd. (Hubei, China). During the pre-trial period, each female donkey was assigned an ear tag to facilitate precise data recording. The test donkey foals and their respective jennies were housed in a specially designed donkey facility, featuring optimal temperature and humidity levels, effective ventilation, and sufficient space. Additionally, the donkey pens were equipped with both sinks and feeding troughs. Throughout the study, a regimen of twice-daily artificial feeding was implemented, with provision for *ad libitum* access to both food and water. Any residual feed was meticulously weighed and documented before the subsequent feeding cycle.

**Table 1:** Composition and nutrient content of basal diets of donkeys.

Ingredients	Content/%	Nutrients	
Wheat straw	42.74	Crude protein/%	6.50
Middlings bran	42.74	Crude fat/%	2.00
Corn	6.41	ADF/%	31.60
Wheat bran	3.42	NDF/%	56.20
Soybean meal	0.85	Acid-insoluble ash/%	7.10
Premix^1^	2.14	Calcium/%	0.79
Premix^2^	1.71	Phosphorus/%	0.20
Total	100.00		

The guaranteed values of premix^1^ composition analysis are: dry base protein + dry base fat ≥ 45%, moisture ≤ 35–45%, crude ash ≤ 9%, crude fiber ≤ 10.5%. The guaranteed values of premix^2^ composition analysis are: *Bacillus subtilis* ≥ 5*107 CFU/g, *Bacillus licheniformis* ≥ 5*107 CFU/g, *Saccharomyces cerevisiae* ≥ 1*106 CFU/g, water ≤ 10%, total arsenic ≤ 5 mg/kg, lead ≤ 10 mg/kg. ADF, Acidic detergent fibers; NDF, neutral detergent fibers.

### 2.3. Sample collection

Blood samples were obtained and subsequently subjected to centrifugation at 3,000 × *g* for 15 min, yielding serum samples which were then meticulously stored at −80°C for subsequent analysis. On the 23rd day of the trial, fecal samples were systematically collected from both female donkeys and donkey foals. Approximately 500 g of fecal material was collected from each female donkey, while donkey foals contributed approximately 100 g each. The collected manure samples from each donkey were divided into 3 sterilized cryopreservation tubes and stored at −80°C for analytical purposes, with any remaining samples preserved within long-arm gloves at the same temperature for further testing.

### 2.4. Growth performance and milk production performance

At the commencement, midpoint, and culmination of the formal trial period, precise fasting weights were recorded before the morning feed. Daily feed consumption data were diligently recorded for each mother-offspring pair, facilitating the calculation of essential metrics, including average daily weight gain, average daily feed intake, and feed weight ratio for donkey foals. These calculations were determined as follows:

- Average daily gain = (last weight − first weight)/test days (Arias et al., 2019)
- Average daily feed intake = total feed intake during the trial period/test days (Torres-Vázquez et al., 2020)

### 2.5. Serum biochemical indexes

Serum biochemical parameters, including total protein (TP), globulin (GLB), albumin (ALB), glucose (GLU), urea nitrogen (BUN), cholesterol (CHOL), alkaline phosphatase (ALP), and other relevant constituents, were meticulously quantified by a method adopted by Zhao et al. (2020) and Shi et al. (2022). For analysis, we used the Pointcare V2 automatic biochemical analyzer manufactured by Tianjin Micro-Nano Core Technology Co., Ltd., with corresponding testing kits also sourced from the same entity.

### 2.6. Immunization indicators

On the 23rd day of the trial, blood samples were obtained, while milk samples were acquired on the 24th day for the assessment of immune parameters, including immunoglobulin A (IgA), immunoglobulin G (IgG), immunoglobulin M (IgM), complement C3, and complement C4. The measurement of these parameters was conducted using methodologies established in prior peer-reviewed publications (Zhao et al., 2020; Du et al., 2022; Shi et al., 2022). The quantification of these immune markers was achieved through the application of the ELISA method, utilizing kits procured from Jiangsu Enzyme Free Industrial Co., Ltd.

### 2.7. Serum metabolomics analysis

Metabolite extraction commenced with the addition of 100 μl of liquid sample to a 1.5 mL centrifuge tube, followed by the introduction of 400 μl of a solution comprised of acetonitrile and methanol in a 1:1 (v/v) ratio, along with 0.02 mg/ml of the internal standard, L-2-chlorophenylalanine. The samples underwent vortex mixing for 30 s and were subsequently subjected to low-temperature sonication for 30 min at 5°C and 40 KHz. Following this step, the samples were placed at −20°C for 30 min to facilitate protein precipitation. Subsequently, the samples were centrifuged for 15 min at 4°C and 13,000 *g*, with the supernatant being carefully removed and evaporated under nitrogen. The dried samples were then reconstituted with 100 μl of a solution comprised of acetonitrile and water in a 1:1 ratio, followed by extraction through low-temperature ultrasonication for 5 min at 5°C and 40 KHz. The samples were once again subjected to centrifugation at 13,000 *g* and 4°C for 10 min. The resulting supernatant was transferred to sample vials in preparation for LC–MS/MS analysis.

The LC–MS/MS analysis was conducted utilizing a Thermo UHPLC-Q Exactive HF-X system, which was equipped with an ACQUITY HSS T3 column (100 mm × 2.1 mm i.d., 1.8 μm, Waters, USA). The mobile phases consisted of a 0.1% formic acid solution in water and acetonitrile, with varying compositions depending on the ionization mode. In the positive ion mode, the gradient ranged from 0 to 100% acetonitrile, while the negative ion mode involved gradients ranging from 5 to 100% acetonitrile. The flow rate was set at 0.40 ml/min, and the column temperature was maintained at 40°C. Mass spectrometric data were acquired using a Thermo UHPLC-Q Exactive HF-X Mass Spectrometer operating in both positive and negative modes, with optimized parameters for ionization and fragmentation. Data were collected across a mass range of 70–1,050 m/z.

### 2.8. Fecal microbiome

#### 2.8.1. DNA extraction and PCR amplification

The extraction of total microbial genomic DNA from fecal samples was carried out employing the E.Z.N.A.® soil DNA Kit, in accordance with the stipulated guidelines of the manufacturer, Omega Bio-tek, Norcross, GA, U.S. Subsequently, the quality and concentration of the extracted DNA were meticulously assessed via 1.0% agarose gel electrophoresis and a NanoDrop® ND-2000 spectrophotometer, manufactured by Thermo Scientific Inc., USA. The purified DNA samples were then preserved at −80°C for subsequent applications. The V3-V4 hypervariable region of the bacterial 16S rRNA gene was specifically targeted for amplification. This amplification process was achieved through the use of primer pairs 338F (5′-ACTCCTACGGGAGGCAGCAG-3′) and 806R (5′-GGACTACHVGGGTWTCTAAT-3′) with product length of 500 bp (Liu et al., 2016), employing the ABI GeneAmp® 9,700 PCR thermocycler (ABI, CA, USA). The PCR reaction mixture consisted of 4 μl 5 × Fast Pfu buffer, 2 μl 2.5 mM dNTPs, 0.8 μl of each primer (5 μm), 0.4 μl Fast Pfu polymerase, 10 ng of template DNA, and ddH2O, with the final volume set at 20 μl. PCR amplification involved the following cycling conditions: initial denaturation at 95°C for 3 min, followed by 27 cycles of denaturation at 95°C for 30 s, annealing at 55°C for 30 s, extension at 72°C for 45 s, and a final single extension step at 72°C for 10 min. Each sample was subjected to triplicate amplification.

#### 2.8.2. Illumina MiSeq sequencing

The purified amplicons, obtained as a result of PCR amplification, were equimolarly pooled and subjected to paired-end sequencing using the Illumina MiSeq PE300 platform. The sequencing process followed standard protocols and was performed by Majorbio Bio-Pharm Technology Co. Ltd. (Shanghai, China).

#### 2.8.3. Data processing

Raw FASTQ files underwent initial demultiplexing using a custom perl script. Subsequently, these files were subjected to quality filtering using fastp version 0.19.6 (Chen et al., 2018) and subsequently merged through FLASH version 1.2.7 (Magoč and Salzberg, 2011). The optimized sequences were then clustered into operational taxonomic units (OTUs) employing UPARSE 7.1 (Stackebrandt and Goebel, 1994; Edgar, 2013), employing a 97% sequence similarity threshold. The most abundant sequence within each OTU was designated as the representative sequence. To mitigate the potential impact of sequencing depth on alpha and beta diversity measurements, the number of 16S rRNA gene sequences from each sample was rarefied to 20,000 sequences. This rarefaction step ensured an average Good’s coverage of 99.09%, respectively.

The taxonomic assignment of each OTU’s representative sequence was executed using the RDP Classifier version 2.2 (Wang et al., 2007), with a confidence threshold of 0.7, against the 16S rRNA gene database (e.g., Silva v138). Additionally, metagenomic functions were predicted via PICRUSt2 (Phylogenetic Investigation of Communities by Reconstruction of Unobserved States; Douglas et al., 2020), leveraging the OTU representative sequences.

### 2.9. Statistical analysis

The data pertaining to growth performance, milk production performance, immunization indicators, and serum biochemical indexes were subjected to rigorous statistical analysis. This analysis included a one-way analysis of variance (ANOVA), performed using SAS version 9.4 (SAS Institute Inc., Cary, NC, USA) software, followed by Duncan’s multiple comparison test. The results are presented as Mean ± SD, with statistically significant differences being established at *p* < 0.05. Probability values ranging from 0.05 to 0.10 were recognized as trends.

For LC/MS raw data preprocessing, Progenesis QI software (Waters Corporation, Milford, USA) was employed to construct a three-dimensional data matrix in CSV format. This matrix encompassed crucial information such as sample data, metabolite names, and mass spectral response intensities. The matrix was subject to further refinement, wherein internal standard peaks and known false positive peaks, including noise, column bleed, and derivatized reagent peaks, were removed. Subsequently, redundant peaks were deduplicated, and peak integration was carried out. The dataset thus obtained, post database search, and was uploaded onto the Majorbio cloud platform (https://cloud.majorbio.com) for comprehensive data analysis. To enhance data quality, variables from quality control (QC) samples with a relative standard deviation (RSD) exceeding 30% were excluded. Furthermore, a log10 logarithmic transformation was applied to these variables, resulting in the final dataset for subsequent analysis. Principal component analysis (PCA) and orthogonal least partial squares discriminant analysis (OPLS-DA) were conducted using the R package “ropls” (Version 1.6.2). Additionally, a 7-cycle interactive validation was performed to evaluate the model’s stability. Metabolites with a VIP > 1 and a *p*-value<0.05, as determined by the Variable Importance in Projection (VIP) obtained from the OPLS-DA model and student’s t-test, were identified as significantly different metabolites.

Sequencing data were predominantly analyzed on the Majorbio Cloud platform (https://cloud.majorbio.com). Based on OTU information, rarefaction curves and alpha diversity indices, such as observed OTUs, Chao1 richness, Shannon indexes, and Good’s coverage, were calculated utilizing Mothur v1.30.1 (Schloss et al., 2009). Principal coordinate analysis (PCoA) using Bray–Curtis dissimilarity was employed to assess the similarity among microbial communities in different samples. The PERMANOVA test was applied to assess both the percentage of variation attributed to the treatment and its statistical significance, using the Vegan v2.5–3 package.

## 3. Results

### 3.1. Growth performance and milk production performance

**Figure 1:**
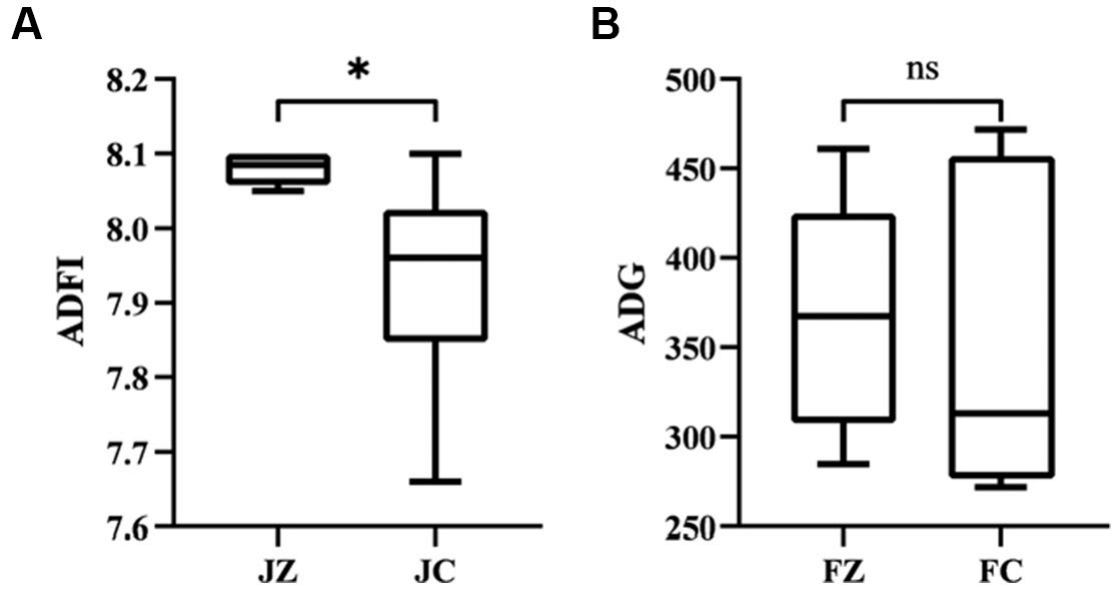
Feed intake of jennies donkeys and daily Weight gain of foals. ADFI, Average daily feed intake; ADG, Average daily gain. FZ, foals in the experimental group; FC, foals in the control group. *, *p* < 0.05. ns, *p* > 0.05.

Figure 1 illustrates the outcomes of growth performance. In comparison to the FC group, the FZ group exhibited a significantly higher average daily feed intake in donkeys (*p* < 0.05). Additionally, the average daily gain in the FZ group was marginally higher than that in the FC group, although this difference did not reach statistical significance (*p* > 0.05). Furthermore, the material weight ratio in the FZ group was significantly lower than that in the FC group (*p* < 0.05).

Table 2 provides an overview of milk production in jennies. The milk production of jennies in the JZ group exceeded that of jennies in the JC group, although the difference was not statistically significant (*p* > 0.05). The analysis of milk composition, encompassing milk protein, lactose, milk fat, and other parameters, revealed no significant differences across the nine indicators (*p* > 0.05).

**Table 2:** Test results for milk yield and milk composition analysis.

Item	JZ	JC	SEM	P
Fat	0.06	0.06	0.012	0.850
Protein	1.24	1.39	0.154	0.497
Lactose	6.47	6.55	0.128	0.688
Total Solid	8.85	8.93	0.137	0.665
Solids-Non-Fat	8.82	8.89	0.156	0.736
Urea	10.92	11.54	0.877	0.625
Lactoferrin	9.18	9.28	0.157	0.667
PFA	0.18	0.18	0.004	0.628
milk yield1	713.3	646.7	94.7	0.63
milk yield2	717.5	648.3	93.4	0.65

PFA, Polyunsaturated fatty acids; SFA, saturated fatty acid. JZ, jennies in the experimental group; JC, jennies in the control group.

### 3.2. Routine animal blood testing

Table 3 presents the results of routine animal blood testing. In comparison to the control group, the experimental group did not exhibit significant differences in levels of TP, GLU, CHOL, ALT, ALP, CRE, WBC, RBC, and HGB (*p* > 0.05). Nevertheless, the GLU levels in colts from the experimental group were significantly lower than those in the control group (*p* < 0.05). Additionally, colts in the experimental group displayed significantly higher ALP levels than those in the control group (*p* < 0.05). Furthermore, foals in the experimental group exhibited significantly lower CRE levels than their counterparts in the control group (*p* < 0.05), while foals in the experimental group displayed significantly higher WBC levels than those in the control group (*p* < 0.05). Importantly, all blood indicators remained within the standard range, affirming the overall health of the donkeys.

**Table 3:** Effects of YPS on blood routine in Dezhou donkeys.

Item	Jenny				Foal			
	JZ	JC	SEM	P	FZ	FC	SEM	P
TP	57.5	56.3	1.28	0.51	55.5	53.7	1.46	0.42
GLU	3.4	3.4	0.17	0.70	4.1	5.1	0.15	0.01
CHOL	1.7	1.8	0.09	0.66	3.0	2.7	0.17	0.35
ALT	8.6	10.6	1.79	0.45	5.4	6.0	0.7	0.35
ALP	123.7	137.0	5.13	0.14	276.4	214.4	17.82	0.04
CRE	72.7	86.7	5.46	0.10	94.5	137.6	7.38	0.03
WBC	7.2	7.1	0.49	0.95	11.2	8	0.86	0.03
RBC	5.4	5.2	0.19	0.61	7.8	6.6	0.4	0.06
HGB	89.5	86.8	2.98	0.54	102.2	87.5	5.02	0.07

TP, Total protein; GLU, glucose; CHOL, cholesterol; ALT, alanine aminotransferase; ALP, alkaline phosphatase; CRE, creatinine; WBC, white blood cell; RBC, red blood cell; HGB, haemoglobin. JZ, jennies in the experimental group; JC, jennies in the control group; FZ, foals in the experimental group; FC, foals in the control group.

**Figure 2:**
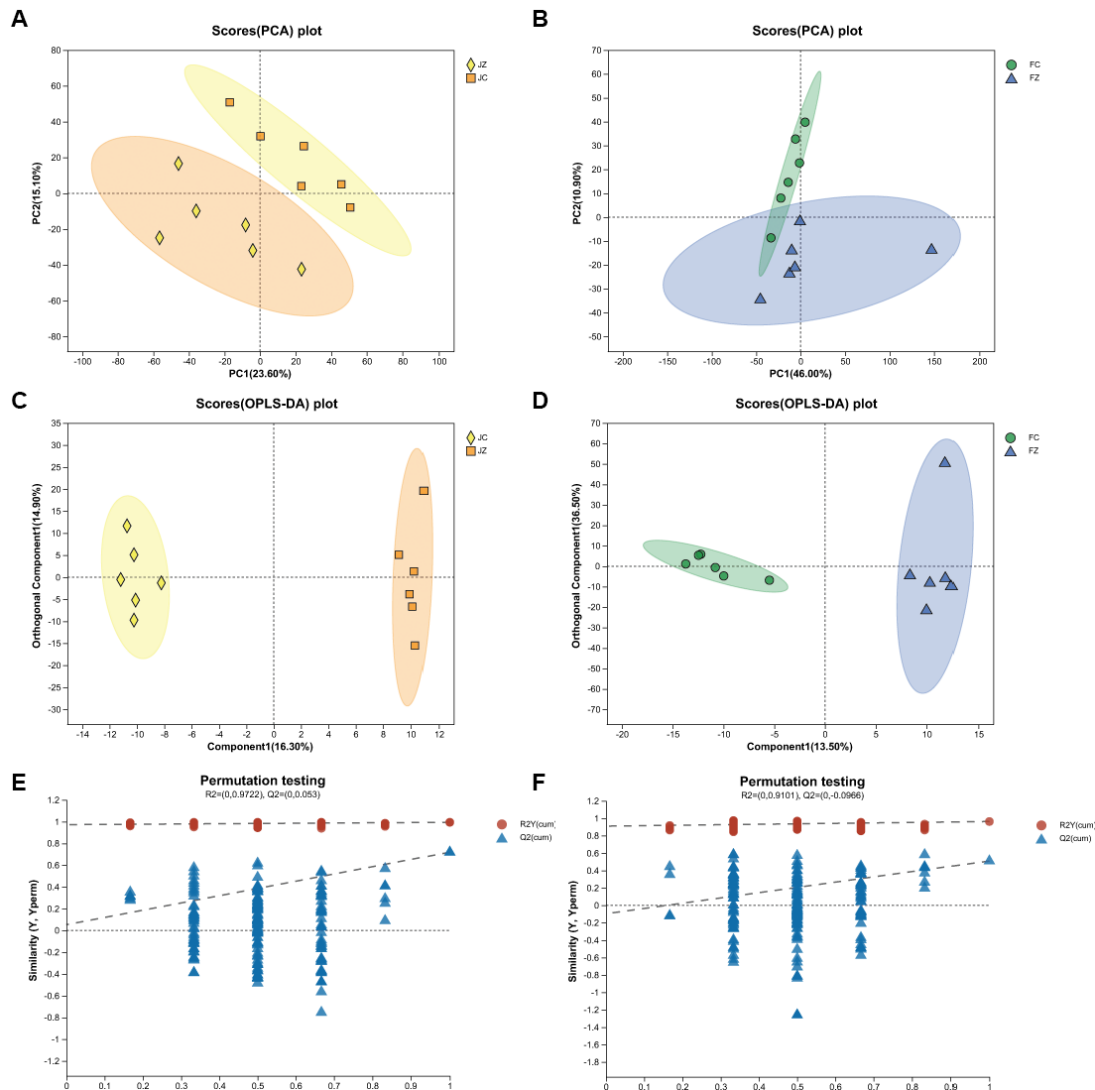
PCA and OPLS-DA scatter plots of jennies and foals. **(A)** PCA of jennies; **(B)** PCA of foals; **(C)** OPLS-DA of jennies; **(D)** OPLS-DA of foals; Panels **(E,F)** are OPLS-DA permutation tests. The R2Y values represents the goodness of fit of the model. The Q2 value represents the predictability of the models.

Principal component analysis (PCA) revealed a distinct separation between the JZ group and the JC group (Figure 2A). However, the PCA data displayed a partial overlap between the FZ group and the FC group (Figure 2B). In contrast, the orthogonal partial least squares discriminant analysis (OPLS-DA) model demonstrated a clear segregation between both the JZ group and the JC group, and the FZ group and the FC group (Figures 2C,D), signifying discernible differences in serum metabolic patterns between the control and YPS groups. Furthermore, all Q2 values exceeded 0.4, confirming the successful establishment of the OPLS-DA models (Figures 2E,F).

**Figure 3:**
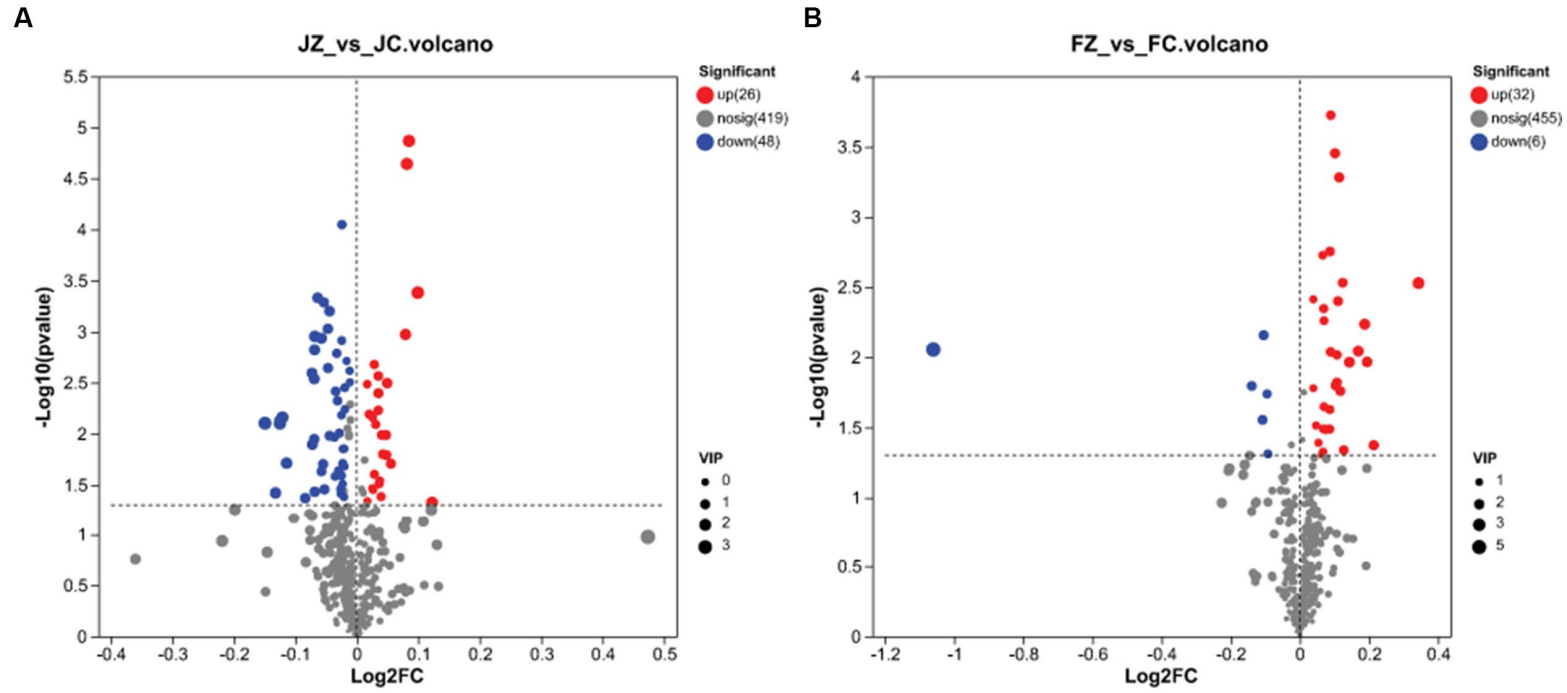
Volcano plots of the identical biomarkers. Red and blue dots indicate upregulated and downregulated metabolites, respectively. Metabolites that showed no difference are sown in gray. **(A)** Volcano plot of the JZ Group compared to the JC Group; **(B)** Volcano plot of the FZ Group compared to the FC Group.

In jennies, 493 metabolites exhibited differential expression (VIP > 1 and *p* < 0.05) between the control and YPS diets. These metabolites were visualized in Figure 3A, with blue compounds representing downregulated biomarkers and red compounds representing upregulated biomarkers. Among these metabolites, 26 were upregulated, while 48 were downregulated. Conversely, in foals, 493 differential metabolites were identified, with 32 being upregulated and 6 displaying moderate downregulation (Figure 3B).

### 3.3. Immunization indicators

**Figure 4:**
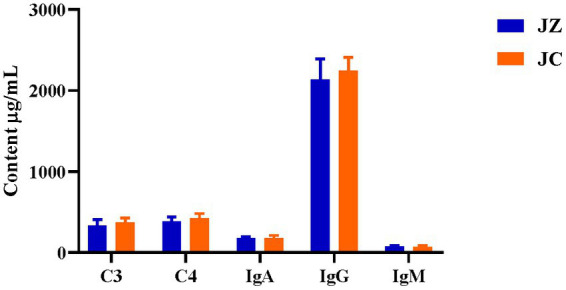
Comparison of immunization indicators in Texas donkey milk. IgA, immunoglobulin A; IgG, immunoglobulin G; IgM, immunoglobulin M; C3, Complement factor 3; C4,Complement factor 4. JZ, Jennies in the experimental group; JC, Jennies in the control group.

Table 4 presents the immunization indicators. In comparison to the JC group, jennies in the JZ group exhibited a significant increase in IgA and IgG levels (*p* < 0.05), with IgM displaying a tendency to increase (*p* = 0.06). However, no significant differences were observed in C3 and C4 levels (*p* > 0.05). In donkey foals, the FZ group displayed significantly higher C4 levels in serum compared to the FC group (*p* < 0.05). Conversely, no significant differences were observed in IgA, IgG, IgM, and C3 levels (*p* > 0.05). When comparing immunization indexes with Texas donkey milk, the FZ group exhibited higher values than the FC group, although these differences were not statistically significant (Figure 4).

**Table 4:** Immunologic indicators in serum.

Item	Jenny				Foal			
	JZ	JC	SEM	*p*	FZ	FC	SEM	*p*
IgA	196.73	143.88	7.10	0.002	175.54	163.02	7.26	0.257
IgG	2393.22	1785.47	89.52	0.003	2144.57	1846.60	117.91	0.112
IgM	84.45	67.13	5.60	0.060	96.16	91.14	7.14	0.653
C3	388.52	344.61	28.68	0.321	423.56	344.58	29.24	0.105
C4	441.11	413.83	17.00	0.320	448.32	378.52	15.94	0.015

IgA, immunoglobulin A; IgG, immunoglobulin G; IgM, immunoglobulin M; C3, Complement factor 3; C4, Complement factor 4. JZ, Jennies in the experimental group; JC, Jennies in the control group; FZ, foals in the experimental group; FC, foals in the control group.

### 3.4. Serum metabolome

Jennies fed the YPS diet exhibited upregulation of 26 metabolites compared to those not receiving additives (*p* < 0.05). Moreover, jennies on the YPS diet displayed downregulation of 48 metabolites compared to jennies in the control group (*p* < 0.05).

**Figure 5:**
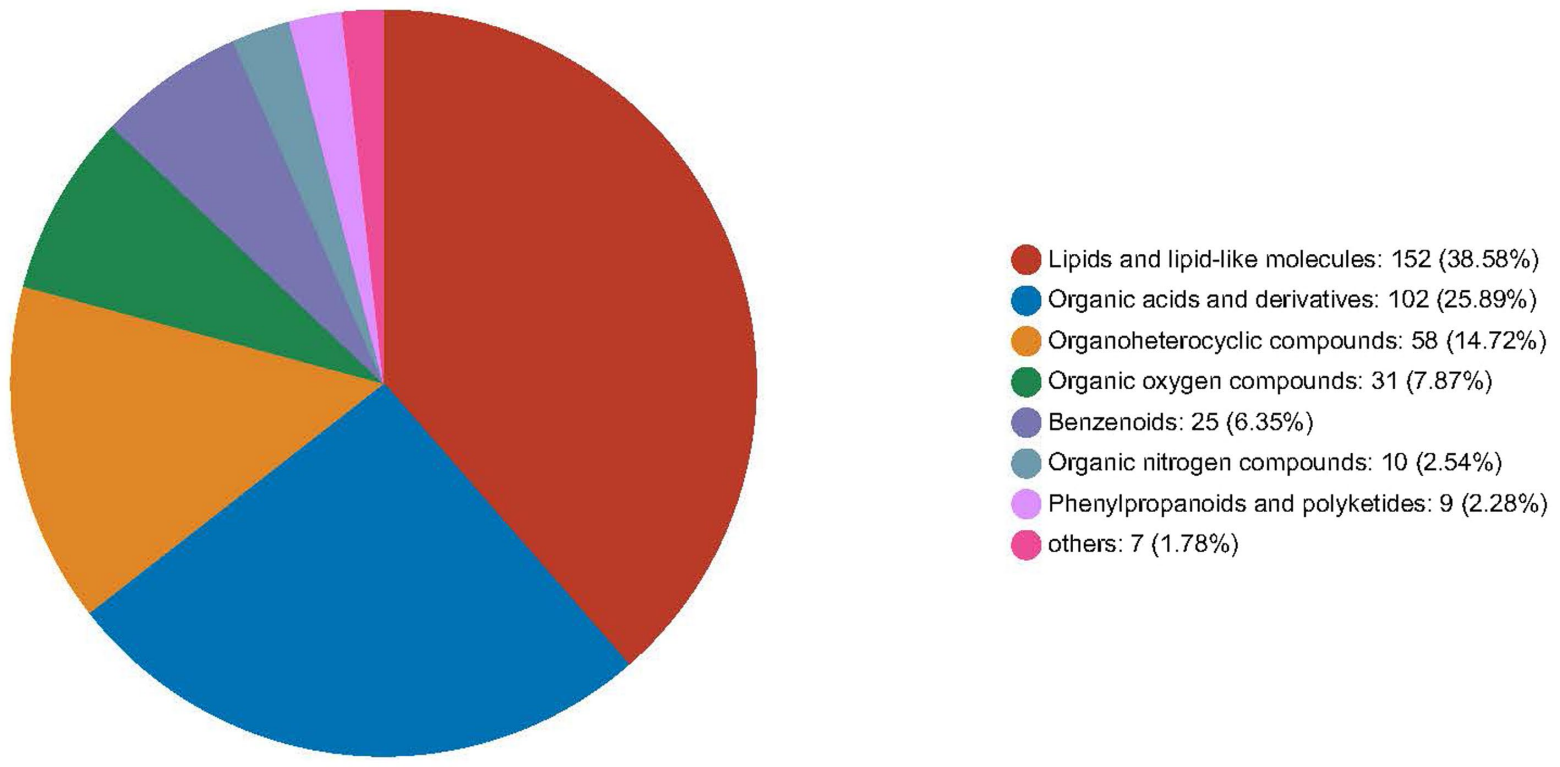
Relative abundance of serum metabolites of donkeys fed control diet and 0.10% yeast polysaccharides (YPS) diet.

**Figure 6:**
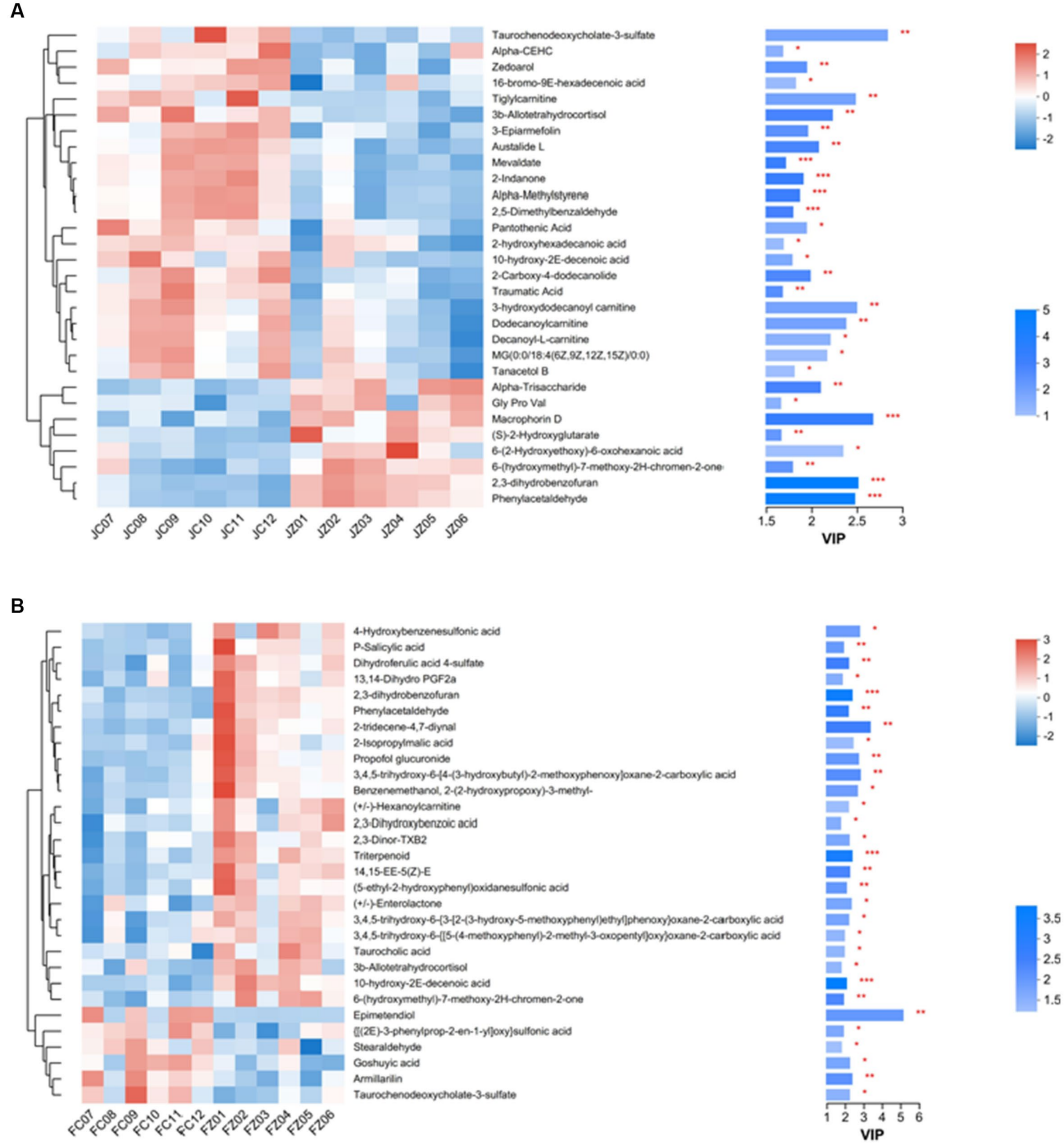
Hierarchical clustering analysis and heat maps of the 30 identified metabolites showing a significant difference between serum samples from donkeys fed control diet and YPS diet. Columns are discrete metabolites, and rows are individual samples, identified for the two diets. The color scale indicates the relative amounts of metabolites: red, higher levels; blue, lower levels; white, unchanged. Levels of significance are defined as *p<0.05, **p<0.01, and ***p<0.001. **(A)** Heat map of the jennies; **(B)** Heat map of the foals.

In foals, the FZ group showed upregulation of 32 serum metabolites and moderate downregulation of 6 metabolites compared to the FC group (*p* < 0.05). Figure 5 provides an overview of the number of HMDB compound metabolites in various categories, with 388 metabolites observed in abundance. These metabolites were categorized into 152 lipids and lipid-like molecules, 102 organic acids and derivatives, 58 organoheterocyclic compounds, 31 organic oxygen compounds, 25 benzenoids, 10 organic nitrogen compounds, 9 phenylpropanoids and polyketides, and 1 other category. A heat map was generated to further investigate the differential metabolites with biological activities within each classification. In jennies (Figure 6A), among the total, 8 compounds were elevated, and 22 compounds were reduced in serum from jennies fed the YPS diet as opposed to the control diet (VIP value>1). These compounds primarily belonged to carbohydrates and carbohydrate conjugates, fatty acids and conjugates, organic acids and derivatives, lipids and lipid-like molecules, organoheterocyclic compounds, and benzene and substituted derivatives. In donkey foals (Figure 6B), 24 compounds were elevated, and 6 were reduced in serum from foals fed the YPS diet compared to the control diet (VIP value>1). These compounds predominantly included benzenoids, organic acids, carbohydrates, and amino acids.

### 3.5. Fecal microbiome

#### 3.5.1. Alpha diversity

**Figure 7:**
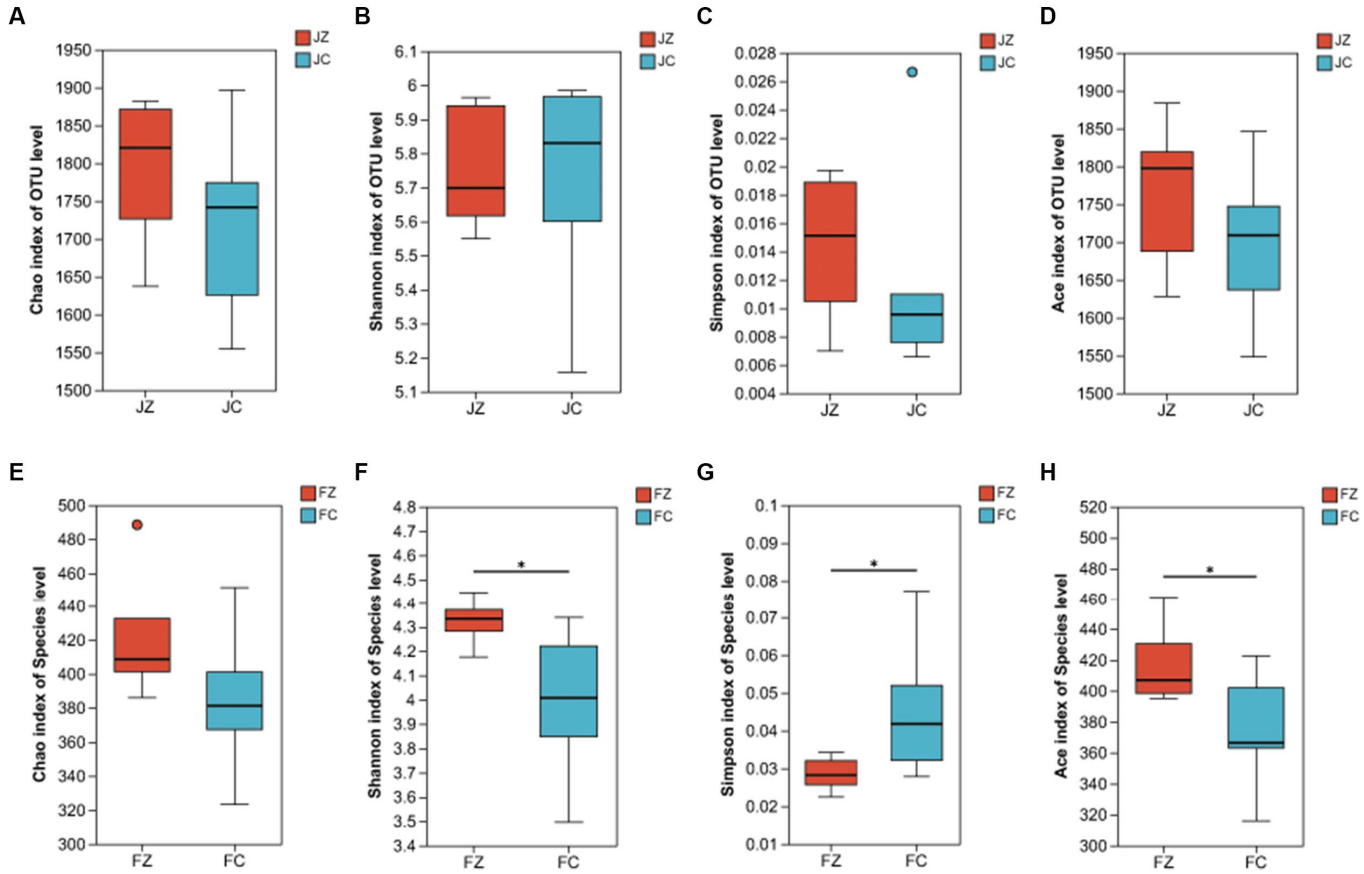
Alpha diversity indices of donkeys in the experimental and control groups. **(A)** Chao index of jennies; **(B)** Shannon index of jennies; **(C)** Simpson index of jennies; **(D)** Ace index of jennies; **(E)** Chao index of foals; **(F)** Shannon index of foals; **(G)** Simpson index of foals; **(H)** Ace index of foals; JZ, jennies in the experimental group; JC, jennies in the control group; FZ, foals in the experimental group; FC, foals in the control group; *, *p*<0.05.

The evaluation of microbial richness and diversity involved the calculation of α-diversity indices, including Chao, Shannon, Simpson, and Ace indices, as depicted in Figure 7. In female donkeys, no statistically significant differences were observed in Sobs, Chao, or Shannon indices between the two groups. Similarly, in jenny ass, there were no significant differences in Chao, Shannon, Simpson, or Ace indices between the two groups (*p* > 0.05). However, in foals, the Shannon and Ace indices of intestinal bacteria in the experimental group were significantly higher than those in the control group (*p* < 0.05), while the Simpson index was lower in the experimental group than in the control group (*p* < 0.05).

#### 3.5.2. Microbial composition

**Figure 8:**
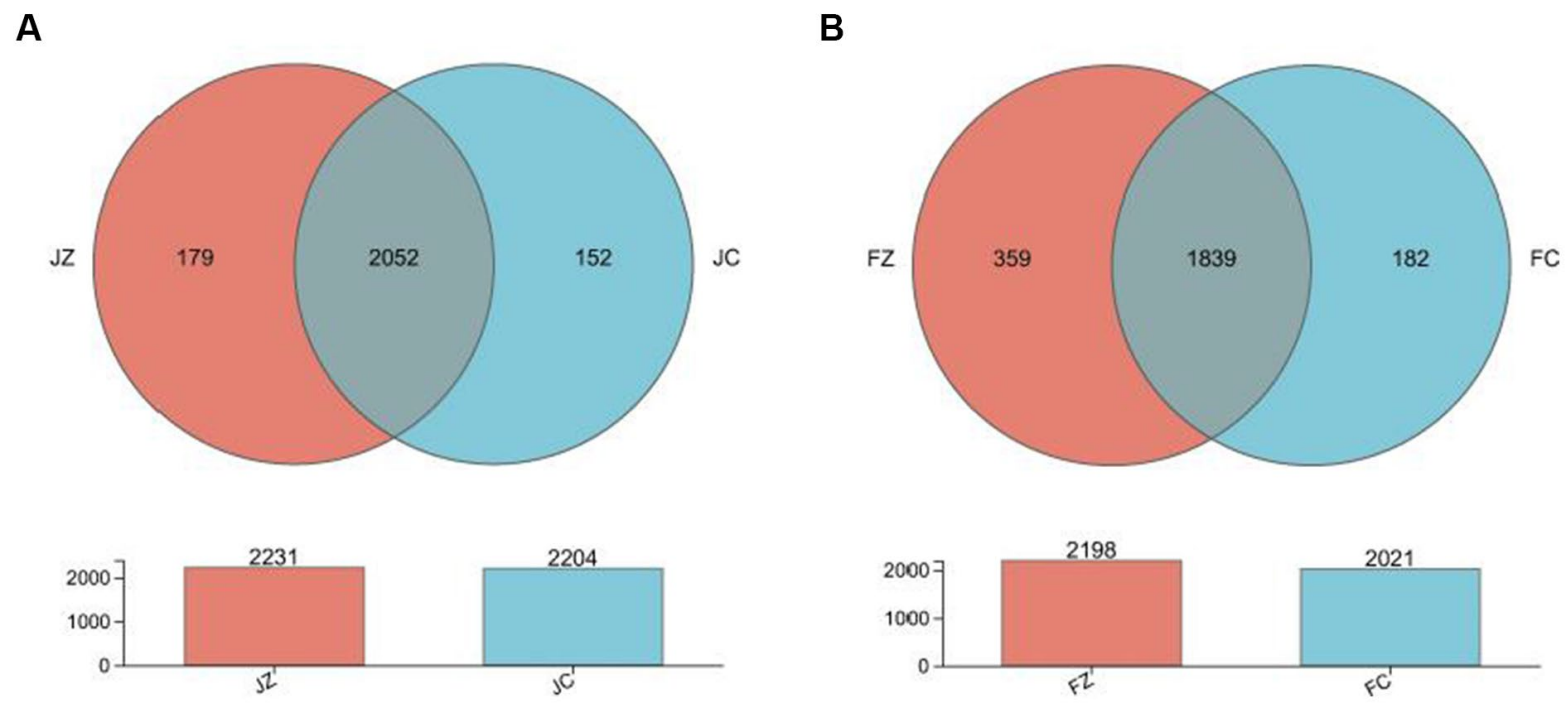
Venn diagram presenting the distribution of intestinal bacteria community OTUs between the experimental and control jennies and between the experimental and control foals. JZ, jennies in the experimental group; JC, jennies in the control group; FZ, foals in the experimental group; FC, foals in the control group.

The Venn diagram presented in Figure 8 illustrates the distribution of bacterial community OTUs. In total, 2,231 and 2,204 OTUs were observed for the intestinal bacteria in group 1 jennies and group 2 jennies, respectively. Additionally, the group 1 jennies shared the intestinal bacteria community, including 2052 OTUs, with the group 2 jennies. In foals, a total of 2,198 and 2021 OTUs were observed for the intestinal bacteria in group 1 and group 2, respectively. Furthermore, the group 1 foals shared 1839 OTUs with the group 2 foals.

**Figure 9:**
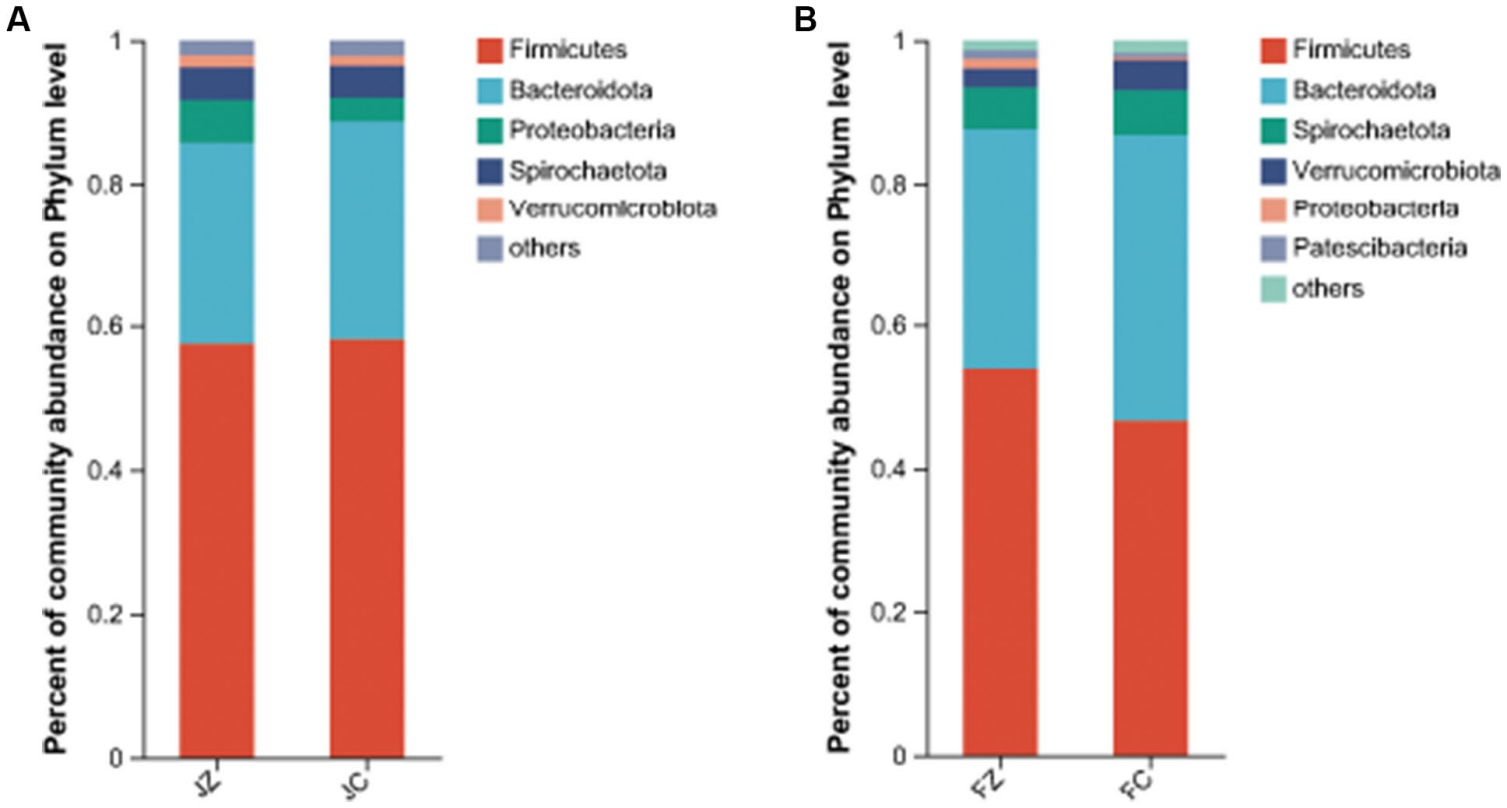
Composition of the predominant intestinal bacteria at phylum level between the experimental and control jennies and between the experimental and control foals (abundance of the phylum ls expressed as %). JZ, jennies in the experimental group, JC, jennies in the control group, FZ, foals in the experimental group, FC, foals m the control group.

Regarding the intestinal bacteria with a relative abundance of more than 1% of the total sequences in at least one of the samples, these were further analyzed (Figure 9). In jennies, the five predominant phyla were *Firmicutes* (57.330.7%), *Proteobacteria* (3.34.5%), and *Verrucomicrobiota* (1.453.9% of the total sequence reads), *Bacteroidota* (33.86.3%), *Verrucomicrobiota* (2.61.4%), and Patescibacteria (0.6 ~ 1.2%).

**Figure 10:**
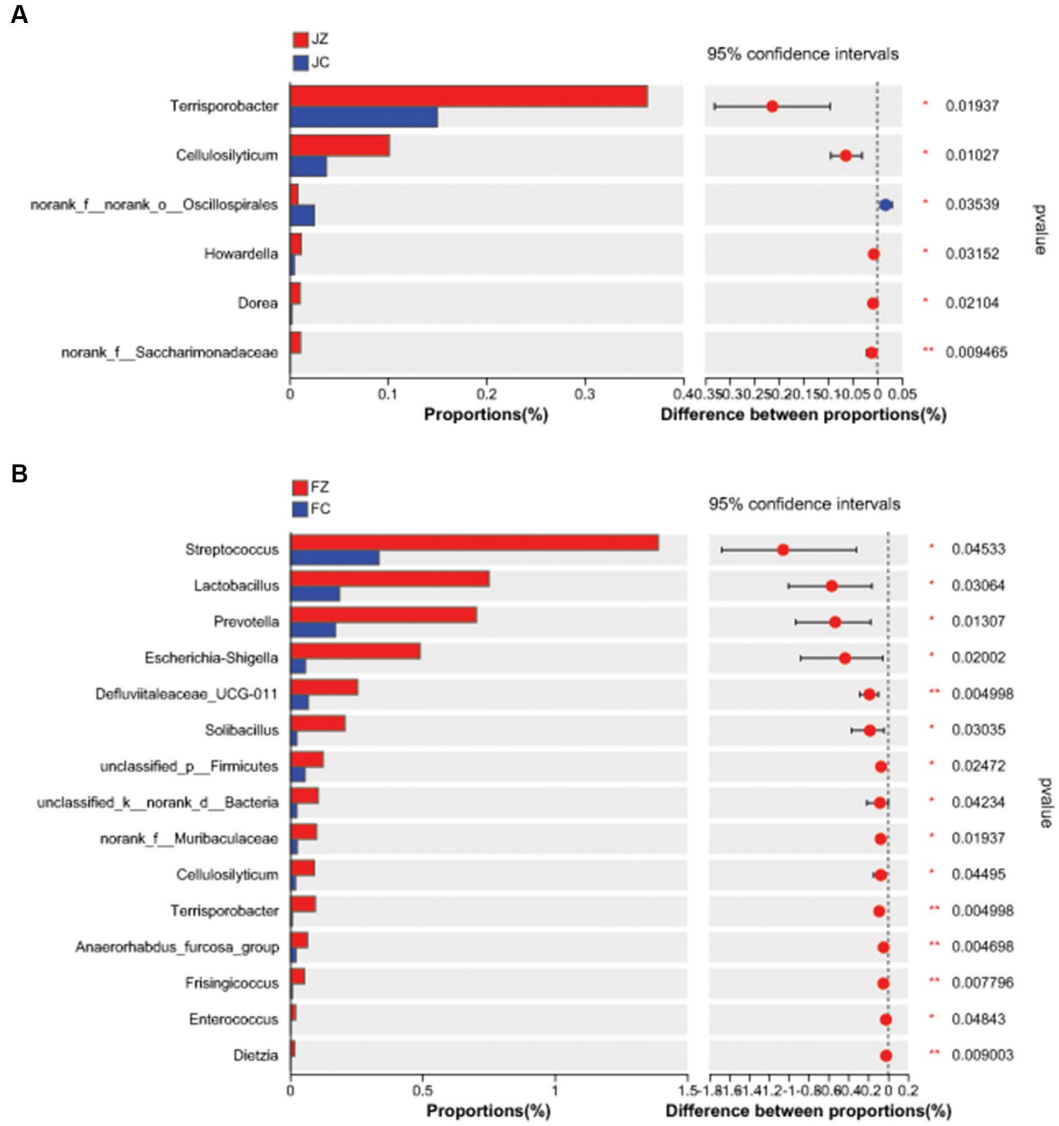
Difference in the predominant intestinal bacteria at genus level between the experimental and control groups. **(A)** Difference in the predominant intestinal bacteria at genus level between the JZ and the JC group; **(B)** Difference in the predominant intestinal bacteria at genus level between the FZ and the FC group; JZ, jennies in the experimental group; JC, jennies in the control group; FZ, foals in the experimental group; FC, foals in the control group.

Differential genera between the two groups were further analyzed (Figure 10). In jennies, the relative abundance of *Terrisporobacter, Cellulosilyticum, Howardella, Dorea,* and *norank_f_Saccharimonadaceae* were significantly greater in the JZ group compared to the JC group (*p* < 0.05). Conversely, the relative abundance of *norank_f_norank_o_Oscillospirales* was significantly lower in the JZ group than in the JC group (*p* < 0.05). In foals, the relative abundance of 15 intestinal bacterial genera, including *Streptococcus, Lactobacillus, Prevotella, Escherichia-Shigella, Defluviitaleaceae_UCG-011, Solibacillus,* and *unclassified_p_Firmicutes,* was greater in the FZ group than in the FC group.

#### 3.5.3. Beta diversity

**Figure 11:**
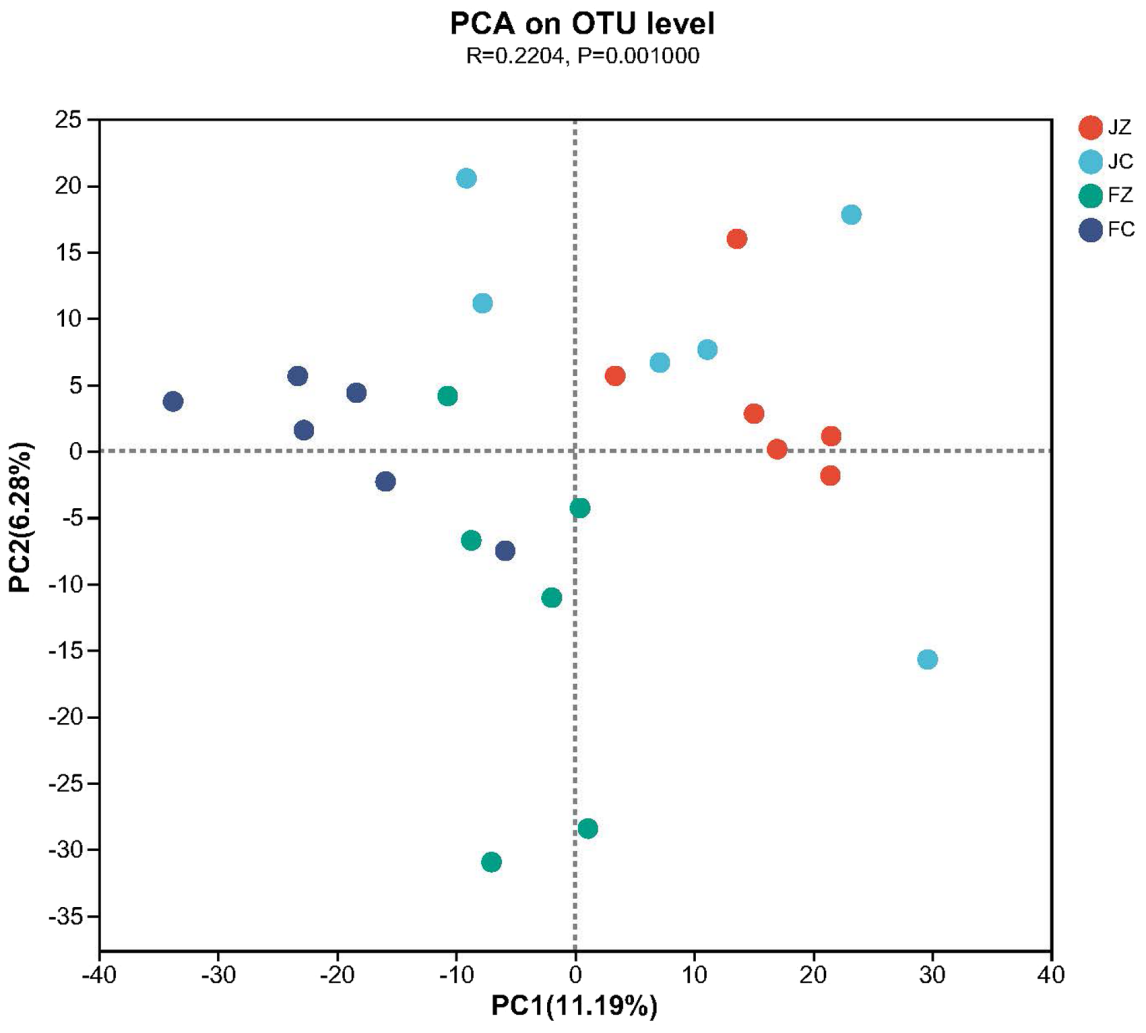
Principal component analysis (PCA) of the intestinal bacteria community composition of the donkeys in experimental and control groups at the OUT level. The Log10 transformed data were used for analysis, and the percentage values given on each axis represent the amount of total variation. PC1, first axis; PC2, second axis. JZ, jennies in the experimental group; JC, jennies in the control group; FZ, foals in the experimental group; FC, foals in the control group.

At the OTU level, beta diversity, as assessed by PCA based on Bray–Curtis dissimilarity, revealed the segregation of intestinal microbiota between the experimental and control groups of donkeys (Figure 11).

## 4. Discussion

### 4.1. Effects of YPS addition on lactating and growth performance

In this study, we investigated the influence of dietary yeast polysaccharide (YPS) supplementation on the growth performance of donkey foals and the milk production performance of Jennies. Our findings revealed that YPS supplementation positively impacted the average feed intake and average daily weight gain of donkey foals, resulting in a reduced feed conversion rate. These results align with previous research, which demonstrated a similar enhancement in the average daily gain and feed conversion rate of Holstein bulls when YPS was added to their feed (Ma et al., 2015). Nonetheless, it’s important to note that studies involving poultry have shown varying effects of YPS on weight gain and feed conversion rate, possibly attributable to differences in factors such as YPS dosage, chicken breeds, and YPS composition (Ahiwe et al., 2019). Furthermore, while our study observed an increase in milk production in female donkeys with YPS supplementation, it is worth mentioning that no prior research has reported similar effects on animal milk production. As the difference in milk production observed in our study was not statistically significant, further investigations are needed to discern the specific impact of YPS on this parameter. Additionally, routine milk analysis revealed no significant differences in milk composition between the two groups of female donkeys.

### 4.2. Effects of YPS addition on immunity

We also assessed the impact of YPS supplementation on immune indices in both jennies and donkey foals, including IgA, IgG, IgM, C3, and C4. Our results demonstrated that YPS inclusion in the diet led to improved immune function in both jennies and donkey foals. This observation aligns with previous research, which has shown that YPS dietary supplementation can significantly elevate serum IgG and IgM concentrations, ultimately enhancing immune function and nutrient digestibility without negatively affecting calf metabolism (Ma et al., 2015). The weaning stage represents a critical period in the overall growth of donkeys, during which foals’ transition from a diet comprising breast milk and feed to pure feed. This transition can result in significant changes in intestinal microflora, potentially leading to stress reactions, including diarrhea, and even mortality in foals (Sun et al., 2023; Zhang et al., 2023). The observed enhancement in immune function in our study could play a vital role in mitigating these stress-related issues.

### 4.3. Effects of YPS addition on donkey metabolism

Given the notable effects of YPS on donkey growth performance and immune function, our study delved into the metabolites and pathways influenced by YPS in donkey foals. The impact of dietary YPS supplementation on serum metabolomics has been minimally explored in previous studies. However, an increasing number of non-targeted metabolomics studies are now being employed to assess the effects of various functional diets on metabolites and pathways in plasma or tissues in animals, contributing to our understanding of diet-nutrition interactions (Tang et al., 2021; Song et al., 2022). In our study, we conducted metabolomic analyses on serum samples from both the 0.1% YPS and control diet groups to identify important differential metabolites and enriched pathways. The robustness and reliability of the obtained metabolomic profiles were supported by the results of the RSD and OPLS-DA models.

Comparing the metabolites with the Human Metabolome Database (HMDB) allowed us to classify them into seven categories. We observed a predominant accumulation of lipids and lipid-like molecules, organic acids and derivatives, organic oxygen compounds, and organic oxygen compounds, with less predominant accumulation of benzenoids, organic nitrogen compounds, phenylpropanoids, polyketides, and other chemical categories. These results are in agreement with previous research that identified lipids and lipid-like molecules, as well as organic acids and derivatives, as significant metabolites in microorganism growth (Chen et al., 2020; Tang et al., 2021). Subsequently, we generated a heat map comparing the control and 0.10% YPS diets to highlight key differential metabolites with biological activities in each classification. Our results indicated that in donkey foals, the most significant differences were observed in the abundance of lipids and lipid-like molecules, including 13,14-Dihydro PGF2a, 2-Isopropylmalic acid, 2,3-Dinor-TXB2, Triterpenoid, Taurocholic acid, 3b-Allotetrahydrocortisol, Epimetendiol, Stearaldehyde, Goshuyic acid, Armillarilin, and Taurochenodeoxycholate-3-sulfate. Specifically, 13,14-Dihydro PGF2a, 2-Isopropylmalic acid, 2,3-Dinor-TXB2, Triterpenoid, Taurocholic acid, and 3b-Allotetrahydrocortisol exhibited increased levels, while the remaining five metabolites showed decreased levels in response to YPS supplementation. It is noteworthy that alterations in lipid and lipid-like molecule metabolism can influence animal growth performance and may be associated with the synthesis of steroid hormones, known growth promoters (Fritsche and Steinhart, 1998).

### 4.4. Effects of YPS addition on donkey gut bacteria

We conducted a comprehensive analysis of the bacterial composition within the intestines of donkeys using 16S rRNA sequencing and identified the dominant genus in both experimental groups. Our results revealed significant differences between the YPS-supplemented group and the control group. The YPS group exhibited significantly higher Ace and Shannon indices, indicating increased diversity and richness in intestinal bacteria. Conversely, the Simpson index was significantly lower in the YPS group, further underscoring the higher diversity and richness in the YPS-treated foals. Dominant phyla in jennies included *Firmicutes, Bacteroidota, Proteobacteria, Spirochaetota,* and *Verrucomicrobiota,* while donkey foals displayed *Firmicutes, Bacteroidota, Spirochaetota, Verrucomicrobiota, Proteobacteria,* and *patescibacteria.* These findings are consistent with Zhang et al. (2022) observations, which investigated fecal microbes in gestating donkeys.

At the genus level, we conducted a detailed analysis of differential genera between the two groups. In jennies, the relative abundance of *Terrisporobacter, Cellulosilyticum*, *Howardella, Dorea,* and *norank_f_Saccharimonadaceae* were significantly higher in the YPS-treated group (JZ) compared to the control group (JC; *p* < 0.05). Conversely, the relative abundance of *norank_f_norank_o_Oscillospirales* was notably lower in JZ compared to JC (*p* < 0.05). Notably, *Terrisporobacter* and *Cellulosilyticum* have been documented as effective cellulose degraders, contributing to enhanced feed digestion (Cai et al., 2010; Cai and Dong, 2010; Niu et al., 2023).

In foals, we observed a higher relative abundance of 15 intestinal bacterial genera in the YPS-supplemented group (FZ) compared to the control group (FC). These genera included *Streptococcus, Lactobacillus, Prevotella, Escherichia-Shigella, Defluviitaleaceae_UCG-011, Solibacillus*, and *unclassified_p_Firmicutes. Lactobacillus* and *Prevotella* are known for their ability to digest and metabolize proteins, carbohydrates, and lipids. Additionally, *Prevotella* significantly increases animal feed intake (Li et al., 2017; Yang et al., 2018; Dao et al., 2021; Gao et al., 2022; Li Y. et al., 2022; Yeoh et al., 2022). In the YPS group, the abundance of *Lactobacillus* and *Prevotella* was significantly higher than in the control group, suggesting a positive impact on feed digestion and conversion rates. These findings are consistent with the research of Zhang et al. (2022), who conducted a study on broiler chickens. In their study, they added licorice polysaccharides to the diet of the chickens, which resulted in increased numbers of *Bifidobacteria* and *Lactobacillus* in the intestinal flora of the chickens. The increased abundance of beneficial bacteria, such as *Bifidobacteria* and *Lactobacillus,* is indicative of improved gut health and digestion efficiency.

## 5. Conclusion

To sum up, our research presents compelling evidence of the beneficial impact of dietary YPS supplementation on various aspects of Dezhou donkeys, including their growth performance, milk production, gut microbiota, and immune function. Additionally, our study reveals that YPS supplementation exerts a regulatory effect on specific serum metabolites, particularly those related to lipids and lipid molecules in both jennies and foals. Through rigorous investigation, we have determined that the optimal level of YPS supplementation for these positive effects is 0.10%. Noteworthy is the significant enhancement in the abundance of bacteria associated with digestive health and intestinal well-being, such as *Lactobacillus* and *Prevotella* in foals and *Terriporobacter* and *Cellulosilyticum* in jennies, resulting from this supplementation. These findings significantly contribute to our comprehension of the metabolic mechanisms responsible for the growth-promoting and immune-regulating effects of dietary YPS supplementation in both jennies and foals. Consequently, YPS emerges as a natural additive with the potential to support the healthy growth and well-being of these animals.

## Data availability statement

The data presented in the study are deposited in the NCBI repository, accession number PRJNA1031603.

## Ethics statement

The animal study was approved by Animal Ethics Committee of Liaocheng University (LC2019-1). The study was conducted in accordance with the local legislation and institutional requirements.

## Author contributions

BH: Conceptualization, Data curation, Formal analysis, Investigation, Methodology, Software, Visualization, Writing – original draft, Writing – review & editing. MK: Data curation, Visualization, Writing – review & editing. YC: Data curation, Formal analysis, Writing – review & editing. HL: Data curation, Methodology, Writing – review & editing. XK: Data curation, Methodology, Writing – review & editing. XW: Data curation, Methodology, Writing – review & editing. WR: Data curation, Methodology, Writing – review & editing. CW: Conceptualization, Data curation, Funding acquisition, Project administration, Resources, Supervision, Validation, Visualization, Writing – original draft, Writing – review & editing. ZZ: Conceptualization, Funding acquisition, Resources, Supervision, Validation, Visualization, Writing – original draft, Writing – review & editing.
